# Codon usage optimization in pluripotent embryonic stem cells

**DOI:** 10.1186/s13059-019-1726-z

**Published:** 2019-06-07

**Authors:** Susanne Bornelöv, Tommaso Selmi, Sophia Flad, Sabine Dietmann, Michaela Frye

**Affiliations:** 1 Department of Genetics, University of Cambridge, Downing Street, Cambridge, CB2 3EH UK; 2 German Cancer Research Center (DKFZ), Im Neuenheimer Feld 280, 69120 Heidelberg, Germany; 3 Wellcome Trust – Medical Research Council Cambridge Stem Cell Institute, University of Cambridge, Tennis Court Road, Cambridge, CB2 1QR UK

**Keywords:** Stem cell self-renewal, Differentiation, Codon bias, tRNA modifications

## Abstract

**Background:**

The uneven use of synonymous codons in the transcriptome regulates the efficiency and fidelity of protein translation rates. Yet, the importance of this codon bias in regulating cell state-specific expression programmes is currently debated. Here, we ask whether different codon usage controls gene expression programmes in self-renewing and differentiating embryonic stem cells.

**Results:**

Using ribosome and transcriptome profiling, we identify distinct codon signatures during human embryonic stem cell differentiation. We find that cell state-specific codon bias is determined by the guanine-cytosine (GC) content of differentially expressed genes. By measuring the codon frequencies at the ribosome active sites interacting with transfer RNAs (tRNA), we further discover that self-renewing cells optimize translation of codons that depend on the inosine tRNA modification in the anticodon wobble position. Accordingly, inosine levels are highest in human pluripotent embryonic stem cells. This effect is conserved in mice and is independent of the differentiation stimulus.

**Conclusions:**

We show that GC content influences cell state-specific mRNA levels, and we reveal how translational mechanisms based on tRNA modifications change codon usage in embryonic stem cells.

**Electronic supplementary material:**

The online version of this article (10.1186/s13059-019-1726-z) contains supplementary material, which is available to authorized users.

## Background

Understanding normal tissue development and disease susceptibility requires the knowledge of mechanisms determining lineage fate decisions in embryonic stem and progenitor cells. While the transcriptional networks governing the naïve and lineage committed states are now increasingly understood [1–3], it remains largely unknown whether and how translational mechanisms contribute to early cell fate decisions.

Translation of mRNA takes place on ribosomes, and distinct sets of tRNAs link each nucleotide triplet in mRNA to a corresponding amino acid. The genetic code is degenerate, because the 64 codons (triplets) encode for only 20 amino acids. Thus, single amino acids are often encoded by multiple synonymous codons that are not used randomly. The uneven use of synonymous codons in the transcriptome is commonly found in different organisms and called codon bias [4]. The selective use of optimal codons that correspond to abundant tRNAs has been suggested to improve translational efficiency by fine tuning translation elongation rates [5–7]. Yet, the importance of codon bias in regulating gene expression programmes remains debated, because other features of the coding sequence, such as guanine-cytosine (GC) content and mRNA secondary structure, also strongly influence translation elongation efficiency [8, 9].

The hundreds of tRNA genes interspersed in the human genome encode only 47 out of the 64 possible tRNA anticodons. Codons lacking a corresponding tRNA are translated via wobble base pairing. While the standard Watson-Crick base pairing is required at the first and second positions, so-called wobbling at the third position can allow otherwise-disfavored base pairing such as between guanosine and uracil [10, 11]. The affinity by which synonymous codons are recognized via wobble base pairing can vary, and the translation kinetics of different codon-anticodon pairs is further influenced by modified nucleotides present in tRNAs [12]. Specifically, position 34 in tRNAs, corresponding to the wobble position, carries a wide range of chemical modifications, including 2’O-methylribose, 5-methylcytidine, 5-methoxycarbonylmethyl, 5-carbamoylmethyl, and their derivatives, as well as direct adenine-to-inosine editing (A34I) [13–15]. These modifications can dramatically alter codon translation rates and for instance adapt protein synthesis to external stress stimuli [16–23].

Whether optimal codon usage through tRNA modifications can regulate cell fate has been unexplored. Therefore, using human embryonic stem cells (hESCs) as a model system, we analysed codon usage optimization during self-renewal and differentiation. We triggered the exit of pluripotency by exposure to retinoic acid [24, 25]. We found that the differentially expressed genes between the cell states were strongly biased by GC content. By analysing the translation kinetics at the ribosomes, we found that self-renewing embryonic stem cells optimized codon usage of specific codons that depended on inosine-modified tRNAs for translation. The differential codon bias was independent of the nature of the differentiation stimulus. In summary, we reveal a codon bias driven by tRNA modifications that defines pluripotent embryonic stem cells.

## Results

### RNA expression and translation highly correlate in self-renewing and differentiating cells

**Fig. 1:**
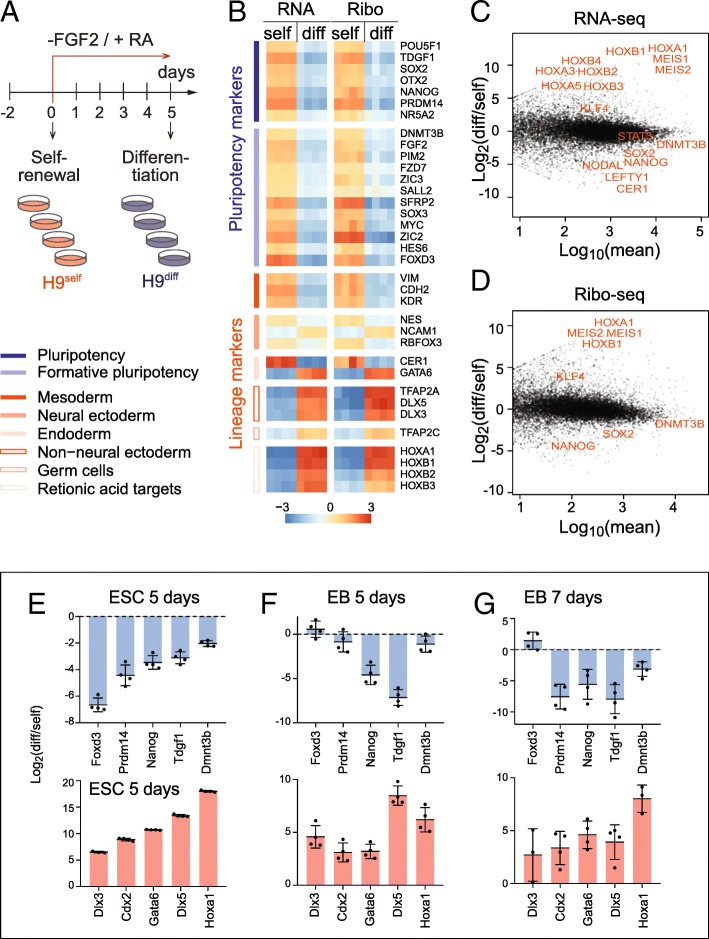
Profiling mRNA expression and translation in self-renewing and differentiating hESCs. **a** Treatment regime to differentiate the hESCs (H9) by removing FGF-2 from and adding retinoic acid (RA) to the culture medium. Four replicates of self-renewing (H9^self^) and differentiating (H9^diff^) hESCs were used for RNA-seq and Ribo-seq analyses. **b** Expression heatmap of pluripotency (blue) and lineage (red) markers measured by RNA-seq and Ribo-seq. Expression is shown as log_2_ difference to the mean. Markers in each group are ordered by decreasing expression. **c**, **d** MA plots showing the log_2_ fold change of differentiated (diff) versus self-renewing (self) hESCs against mean expression in either RNA-seq (**c**) or Ribo-seq (**d**). Examples of significant genes related to differentiation are shown in red. **e–g** RT-qPCR confirming similar change in RNA levels of pluripotency markers (upper panels) and lineage markers (lower panels) in hESCs RA-differentiated for 5 days (**e**) and in embryoid bodies grown for 5 days (**f**) or 7 days (**g**). Shown is the mean (*n* = 3–4). Error bars s.d.

To determine mRNA expression and translation levels in self-renewing and differentiating human embryonic stem cells (hESCs), we performed RNA-sequencing (RNA-seq) and ribosome footprint profiling (Ribo-seq). We induced the exit of pluripotency of hESCs by removing the growth factor FGF-2 and exposing the cells to retinoic acid (RA), one of the best characterized stimuli to induce early embryonic differentiation (Fig. 1a) [26, 27].

After sequencing, we determined the quality of the Ribo-seq data. The library composition of self-renewing and differentiating hESCs was comparable and contained a considerable number of reads mapping to ribosomal RNAs (rRNA), transfer RNAs (tRNAs), and adapter dimers, as expected (Additional file 1: Figure S1A). We found a large number of genes differentially expressed (Additional file 1: Figure S1B; Additional file 2: Table S1). The biological replicates were highly correlated (*r* = 0.98–1.00) and clustered together (Additional file 1: Figure S1C). In contrast, the correlation between self-renewing and differentiating samples were much lower (*r* = 0.70–0.77 in Ribo-seq; *r* = 0.85–0.88 in RNA-seq). Both the read length distribution and the strong reading frame periodicity for reads of 27 to 29 nucleotides in length confirmed sufficient coverage of mRNAs to analyse translation at single codon resolution (Additional file 1: Figure S1D-F) [28]. Metagene analysis further confirmed a strong enrichment of reads over the coding sequences, with only a small number of reads mapping to the 5′ UTRs and nearly no reads at the 3′ UTRs (Additional file 1: Figure S1G). Finally, the differentially abundant genes in the Ribo-seq and RNA-seq datasets highly correlated (Additional file 1: Figure S1H), suggesting that transcriptional mechanisms might be the dominant factor changing mRNA levels upon exit from pluripotency [29].

Induction of stem cell differentiation using RA in the absence of FGF-2 induced transcripts of all primary germ layers (Additional file 3: Table S2). Both RNA-seq and Ribo-seq confirmed the existence of heterogeneous cell populations expressing markers of ectoderm, endoderm, and mesoderm, while pluripotency markers were consistently repressed (Fig. 1b-d) [30–33]. The upregulated genes further included the *Hox* family, which is known to be regulated through RA-signalling in early embryonic development [34]. To further confirm that we efficiently differentiated the hESCs, we also grew hESCs in suspension to induce their differentiation into embryoid bodies (EBs) for 5 and 7 days [35]. The change of mRNA levels of pluripotency and lineage markers were comparable to RA-induced differentiation (Fig. 1e–g). Thus, RA-treated hESCs exited the pluripotent state and underwent cell differentiation.

### Codon composition of cell state-specific mRNAs is biased towards GC content

**Fig. 2:**
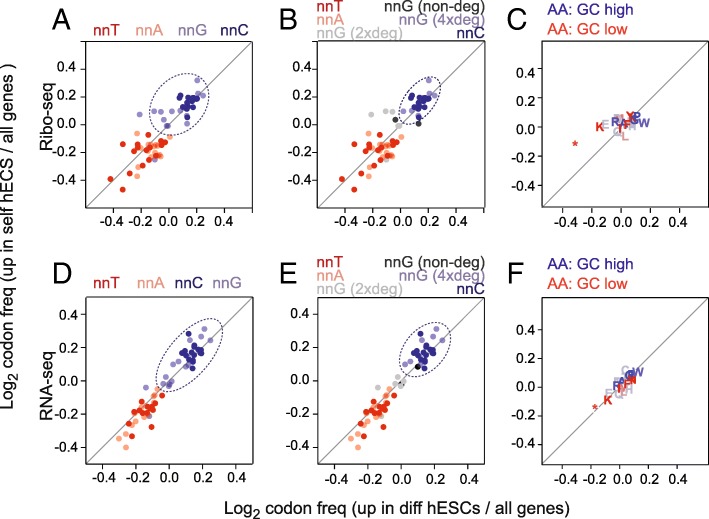
Genomic GC content influences codon usage. **a–f** Overview of codon (**a**, **b**, **d**, **e**) and amino acid (**c**, **f**) enrichment in differentially expressed genes measured by Ribo-seq (**a–c**) and RNA-seq (**d–f**). Enrichment was calculated as log_2_ fold change of codon or amino acid frequency in differentiation or self-renewal genes relative to all genes. Codons are colour coded according to their third nucleotide (**a**, **d**) and are further separated by *nn*G codon degeneracy (**b**, **e**). Dotted circles highlight the most enriched codons in response to the retinoic stimulus

We next asked whether self-renewing and differentiating cells optimized their translational programmes by using cell state-specific codons. First, we selected all well-annotated coding sequences from the consensus coding sequence project [36]. Then, we calculated the relative codon frequency of each gene; thereby, each gene was represented as vector of 64 codon frequencies. Using our data, we defined two groups of genes: (i) significantly upregulated genes in self-renewing hESCs and (ii) significantly upregulated genes in differentiating hESCs, and then calculated the overall codon usage compared to all genes (Fig. 2).

We reasoned that if codon bias contributed to altering mRNA translation between self-renewing and differentiating cells, then it would be most likely to occur at the wobble positions due to the relaxed base pairing and the degeneracy of the genetic code. Therefore, we subdivided the codons according to the base at the wobble positions (*nn*A, *nn*T, *nn*C, and *nn*G) (Fig. 2a). The codon enrichment ratios were highly similar for both the upregulated genes in self-renewing and differentiating hESCs (Fig. 2a). Genes that changed most in expression upon retinoic acid treatment were commonly enriched in G and C at the wobble position (Fig. 2a; dotted circle) and depleted in A and T. While *nn*C codons clustered closely together, *nn*G codon were more diffuse; this was correlated to the codon degeneracy. Only four-fold degenerate *nn*G codons clustered closely with *nn*C codons (Fig. 2b). Next, we asked whether the codon bias correlated with a different amino acid usage in the two cell states. However, neither up- nor downregulated genes showed an enrichment for a specific amino acid usage (Fig. 2c).

We considered the possibility that the enrichment of *nn*C and *nn*G codons was driven by the translational machinery. However, we observed a similar distribution of codon usage in the upregulated genes of both cell states obtained by RNA-seq (Fig. 2d–f). Thus, our data indicated that the difference in codon usage in self-renewing and differentiating hESCs was already present at the mRNA level. Possible underlying mechanisms causing the distinct codon usage in self-renewing and differentiating hESCs are for example DNA transcription or RNA decay.

The use of optimal codons can enhance mRNA stability and translation, and this effect is evolutionary conserved [37–40]. To address whether optimal codons enhanced mRNA stability in the two cell states, we calculated the relative enrichment of stabilizing and destabilizing codons (Additional file 1: Figure S2A) using a codon classification made in zebrafish [39]. The upregulated mRNAs in both conditions showed a slight depletion of strongly destabilizing codons (Additional file 1: Figure S2B). When we measured the difference between the self-renewing and differentiating genes, we found that upregulated mRNAs in self-renewing hESCs contained significantly fewer destabilizing codons and slightly more highly stabilizing codons (Additional file 1: Figure S2B; right-hand panels). The association of GC content at the third nucleotide of a codon to high levels of mRNAs was however more pronounced than the association to stabilizing and destabilizing codons (Additional file 1: Figure S2C, D). Together, these data demonstrated that abundant mRNAs in both the self-renewing and differentiated state were biased towards a higher GC content, but that in addition, codon optimality contributed to the codon usage differences between the two cell states.

### GC content defines groups of genes with common function

**Fig. 3:**
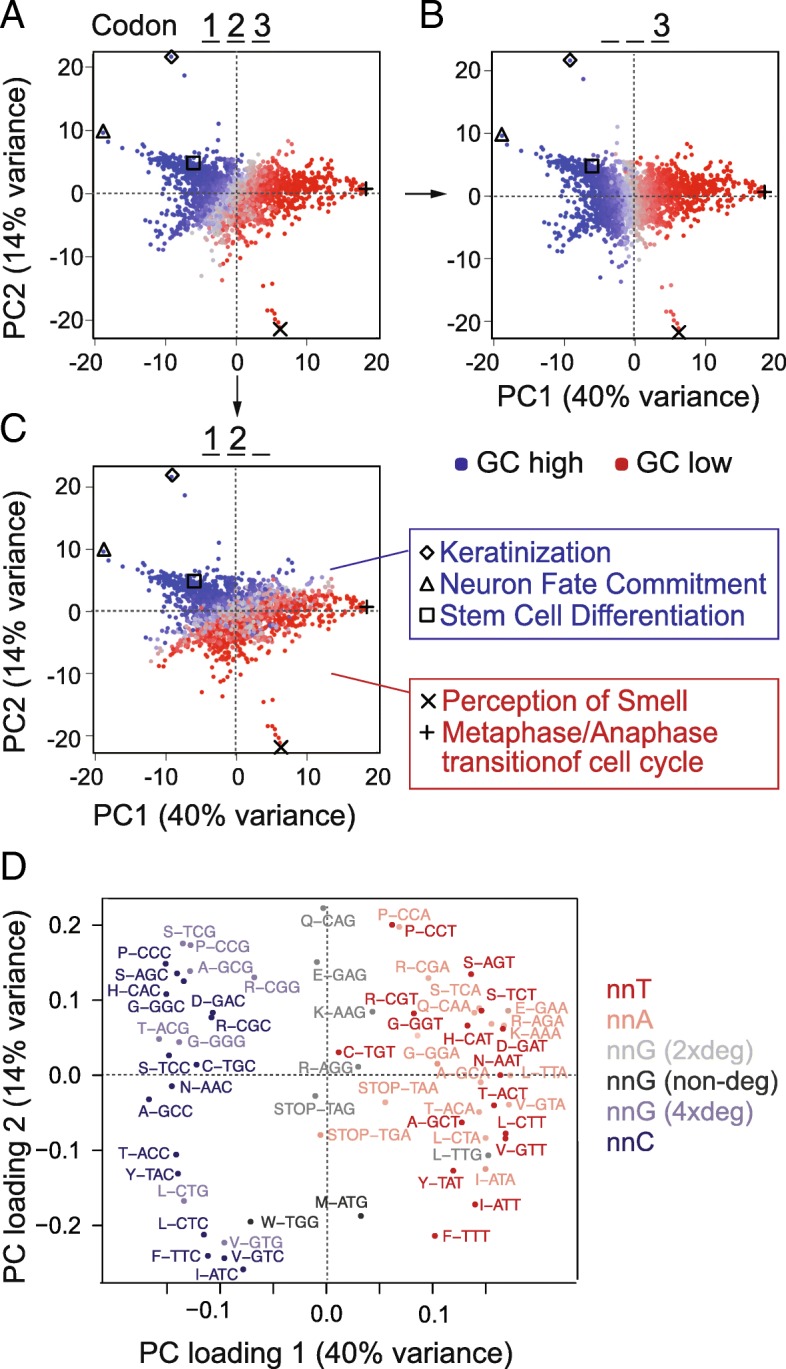
Genes from different GO categories show different codon frequencies. **a–c** Codon frequencies were calculated per Gene Ontology (GO) term, and PCA was used to reveal a global separation into high and low GC genes (**a**). GO terms are coloured according to total GC (**a**), GC at the third codon position (**b**), or at the first and second position (**c**). Examples of GO terms are listed next to the graphs. **d** PCA loadings plot identifying the codons contributing the location of the GOs in **a–c**. Codons are colour coded by third position base and/or codon degeneracy. The separation across the *x*-axis is comparable to the separation across the diagonal in Fig. 2a, b, d, e

Underlying GC content can substantially contribute to codon usage differences among different cell states [9, 41, 42]. We therefore analysed codon usage in genes belonging to various functional categories, including those deployed during self-renewal and differentiation of hESCs. We selected all well-annotated coding sequences and grouped them according to their Gene Ontology (GO) terms. We calculated the average of codon frequencies per GO term and used principal component analysis (PCA) to reveal the underlying sources of variance. The GO terms were primarily separated by GC content (Fig. 3a); indeed, the first principal component closely corresponded to the GC content of the third codon base (Fig. 3b). The second principal component reflected the GC content at the first and second bases (Fig. 3c). Accordingly, genes belonging to similar GO terms such as cell differentiation and neuron fate commitment had similar and high GC content (blue) (Fig. 3c; right panels).

It was unexpected that all three codon positions separated the GO terms according to GC content. While the third and variable base position of codons can be readily varied to optimize translation rates without impacting the encoded amino acids [5], changes in the first and second positions almost always result in a different amino acid. Nevertheless, we found that genes belonging to different functional categories were characterized by different codon usage and that this correlated with GC content.

Using a PCA loading plot, we identified the codons driving the global separation by GC content (Fig. 3d). Codons with cytosine at the third position contributed to the left and codons with adenosine or thymidine at the third position contributed to the right of the plot. As described for our own differentiation data (Fig. 2b, e), only four-fold degenerate codons with guanosine at the third position contributed to the left of the PCA plots (Fig. 3d). The high level of similarity between the global PCA analysis of all genes and our differentiation experiment suggested that genes with similar functions share similar inherent genomic features. In conclusion, genomic GC content influences codon choice in the differentiation gene expression programme.

### Self-renewing ESCs share a common codon signature at the ribosome A- and P-sites

While GC content was one factor for codon usage optimization, protein translation efficiency is likely to be regulated by additional factors. The translation kinetics of codon-anticodon pairs in the ribosome are complex and determined by the availability and binding affinity of matching and mismatching tRNAs [5, 43, 44]. In part, the translation rate is directed by the competition between near-cognate and cognate aminoacyl-tRNAs entering the ribosome. Therefore, we asked whether self-renewing and differentiating hESCs showed differential codon stalling at the active sites of the ribosome.

**Fig. 4:**
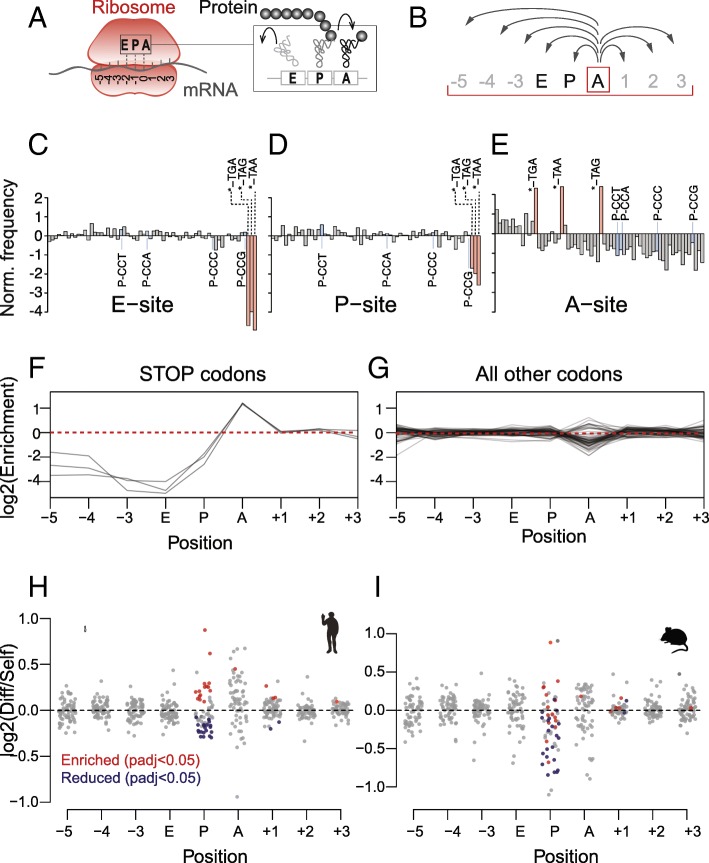
Distinct codon signature at the ribosome A- and P-sites in self-renewing ESCs. **a** Illustration of a ribosome binding to mRNA protecting at least nine codons (position − 5 to + 3) from degradation by the RNase digestion. Positions − 2, − 1, and 0 correspond to the E-, P-, and A-site that host the charged tRNA. **b** The raw codon frequency was calculated for each specific codon (e.g. A-site) and normalized to the mean frequency across all nine positions. Calculations were done separately for self-renewing and differentiating samples. **c–e** Codon enrichment at the E- (**c**), P- (**d**), and A-sites (**e**). Stop codons are highlighted in red, and proline codons are highlighted in blue. The codons are sorted according to their frequency (shown in Additional file 1: Figure S3A-C). **f**, **g** Codon enrichment of stop (**f**) or all other codons (**g**) across all ribosome-protected codons. **h** Log_2_ fold change of normalized codon usage in differentiated versus self-renewing hESCs across all ribosome-protected codons. Significantly different codons (Welch’s *t*-test; FDR correction) are marked in red (enriched) and blue (reduced) and occur mainly at the P-site. **i** Log_2_ fold change of normalized codon usage in mouse differentiated (embryoid bodies) versus self-renewing embryonic stem cells (mESCs). Blue and red dots represent the significantly changed codon shown in **h**

During translation, only three codons actively pair with tRNAs, known as the ribosome E-, P-, and A-sites (Fig. 4a). Cognate aminoacyl-tRNAs enter the ribosome at the A-site. After transfer of the polypeptide chain to the tRNA, the tRNA moves from the P- to the E-site and is then released from the ribosome. Although the ribosome only actively engages with three codons within an RNA, it covers more than 27 nucleotides (Fig. 4a). Due to the three-nucleotide periodicity reflecting the triplet nature of the genetic code (Additional file 1: Figure S1E, F) [45], the actively bound codons at the E-, P-, and A-sites can be calculated by counting nucleotide triplets from the 3′ end of the read fragments. We numbered the ribosome-protected codons from − 5 to + 3, with 0 corresponding to the A-site (Fig. 4a). We first calculated the codon frequencies per footprint position. To do so, we compared the relative frequency of a codon at the ribosome P- (or A-) site to the frequency of this codon in all Ribo-seq reads from the sample.

As expected, some codons occurred more frequently than others (Additional file 1: Figure S3A-C). Since the codon frequencies at the positions flanking the tRNA-bound sites (− 5 to − 3, and + 1 to + 3) are expected to follow the genomic codon distribution, they served as a negative control. We then normalized the raw frequencies to the frequency across all nine positions (Fig. 4b). This will correct for differences in mRNA abundance, and neither transcriptional nor differences in mRNA decay should affect the following analysis. The strong enrichment of stop codons at the A-site and their depletion at the P- and E-sites validated this approach (Fig. 4c–e). Although Proline incorporates more slowly in translation [46], its codons were not strongly enriched at the A-, P-, or E-site (Fig. 4c–e). When we compared the enrichment of codon frequencies along all sites in the ribosome, we found the highest variation at the A-site (Fig. 4f, g), indicating that the stalling at the A-site was rate limiting for translation elongation [47, 48].

Next, we tested whether the normalized codon frequencies differed in self-renewing and differentiating hESCs (Fig. 4h). We observed differences at both the A- and the P-sites, yet most significant differences occurred at the P-site (Fig. 4h; Additional file 1: Figure S3D). To test whether the codon usage differences in human ESCs was an evolutionarily conserved feature of translational control, we performed similar analyses using published Ribo-seq and RNA-seq data obtained from self-renewing and differentiating mouse embryonic stem cells (mESCs) [49]. The exit of pluripotency and differentiation of the mESCs was induced using two well-established methods: the withdrawal of leukemia inhibitory factor (LIF) from the culture medium and by growing the mESCs as embryoid bodies (EBs) [50–52]. As described for hESCs, we excluded rRNA and tRNA reads and selected read lengths with high reading frame periodicity (Additional file 1: Figure S4A-E). Again, most differences occurred at the A- and P-sites (Fig. 4i). Although the mouse data were noisier, the significantly depleted codons at the P-site in differentiated hESCs were often also less common in differentiated mESCs (Fig. 4h, i; Additional file 1: Figure S4F; blue dots). Thus, we found a distinct set of codons with a higher likelihood of being stalled at the ribosome P-site in mouse and human self-renewing embryonic stem cells.

### A prominent self-renewing codon signature is hetADAT-sensitive

**Fig. 5:**
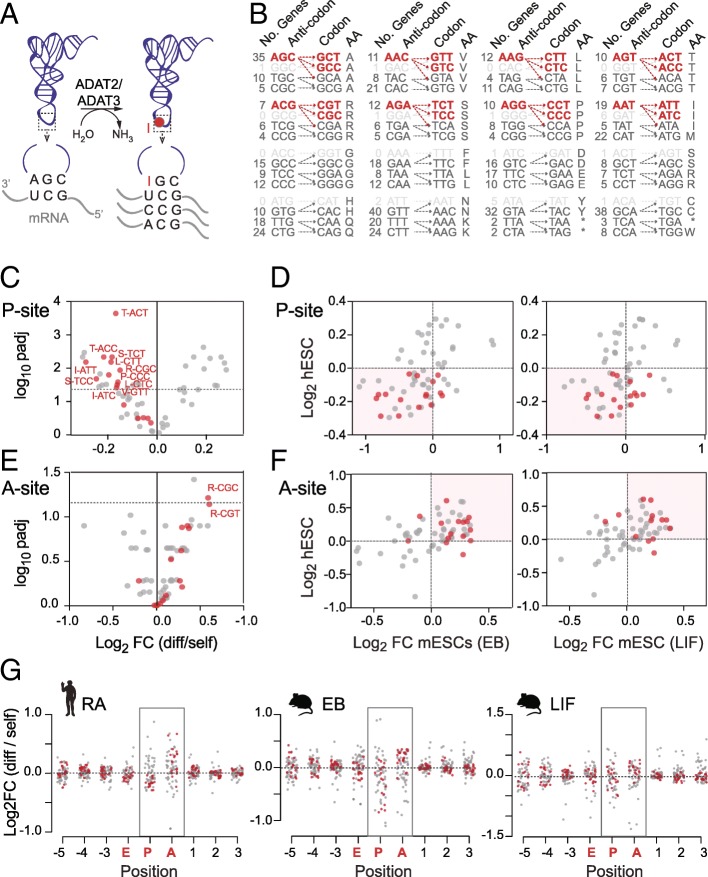
Distinct codon usage signature in pluripotent cells are hetADAT-sensitive codons. **a** Illustration of hetADAT (ADAT2 and ADAT3)-mediated adenosine-to-inosine tRNA editing at position 34 in the anticodon loop and the codons read by unmodified and modified tRNAs. **b** HetADAT-dependent codons (red) that sometimes are lacking corresponding tRNA genes (light gray). **c–f** Volcano plot (**c**, **e**) showing significance versus codon usage fold change at the ribosome P-site (**c**) and A-site (**e**) in differentiated versus self-renewing hESCs. HetADAT-dependent codons are highlighted in red. Correlation (**d**, **f**) between log_2_ fold change of codon usage frequencies in differentiated human (hESCs) with differentiated mouse (mESCs) embryonic stem cells through embryoid body culture (EB; left-hand panel) or by removal of LIF (right-hand panel) at the P-site (**d**) and A-site (**f**). **g** Log_2_ fold change of codon usage frequency across all sites protected by the ribosome in human (left-hand panel) and mouse ESCs differentiated through EB culture (middle panel) or removal of LIF (right-hand panel). HetADAT-dependent codons are highlighted in red

A key factor modulating codon translation is tRNA selection [19]. To enhance codon binding, the tRNA wobble nucleoside (position 34) frequently carries chemical modifications [53]. One such prominent and essential tRNA modification is wobble inosine [54, 55]. Inosine is formed by a deamination reaction of adenosine and catalyzed by the heterodimeric enzyme adenosine deaminase acting on tRNA (hetADAT) composed of two subunits ADAT2 and ADAT3 (Fig. 5a) [56]. While tRNAs carrying adenosine at position 34 (A34) pair with uracil, inosine (I34) is capable of pairing with adenosine, uracil, and cytosine and thereby enhances translation efficiency (Fig. 5a) [11, 57]. In eukaryotes, the modification is present in eight different tRNAs that recognize the eight *nn*T codons. Inosine in tRNAs is required to translate *nn*C codons that have none or few tRNA gene copies (Fig. 5b) [58]. In principle, the same tRNAs can also bind eight *nn*A codons, yet these codons are also translated by their own complementary tRNAs.

We noticed that codons showing significant differences in self-renewing hESCs were often translated by inosine-modified tRNAs (Fig. 5c). All *nn*T and *nn*C codons that depended on inosine tRNA for translation occurred less often at the P-site in differentiated hESCs (Fig. 5c). Moreover, these differential codon frequencies highly correlated between human and mouse (Fig. 5c). Thus, mouse and human differentiating ESCs showed a depletion of hetADAT-dependent codons at the P-site. As described above, due to the high variability of codon frequencies at the A-site, we were unable to identify significant changes (Fig. 4h). However, when we now plotted the A34I-dependent codons, we found the vast majority of them enriched at the A-site in both mouse and human differentiating ESCs (Fig. 5e, f; Additional file 4: Table S3a,b). Finally, we confirmed that this distribution was specific to the A- and P-sites and did not occur at any other site in the ribosome (Fig. 5g). In summary, we found that in self-renewing ESCs hetADAT-dependent codons occurred less often at the ribosome A-site but more often at the P-site when compared to differentiated ESCs.

The time it takes the ribosome to ensure that a tRNA in its A-site is indeed a cognate tRNA for the codon, is considered rate limiting for translation elongation [59, 60]. In addition, whenever a ribosome is bound to an mRNA waiting for a cognate tRNA at its A-site, a tRNA is already bound to its P-site, attached to the growing polypeptide chain. Thus, the lower frequency of hetADAT-dependent codons at the A-site indicated their faster translocation to the P-site in self-renewing ESCs.

### hetADAT regulates codon usage in self-renewing embryonic stem cells

**Fig. 6:**
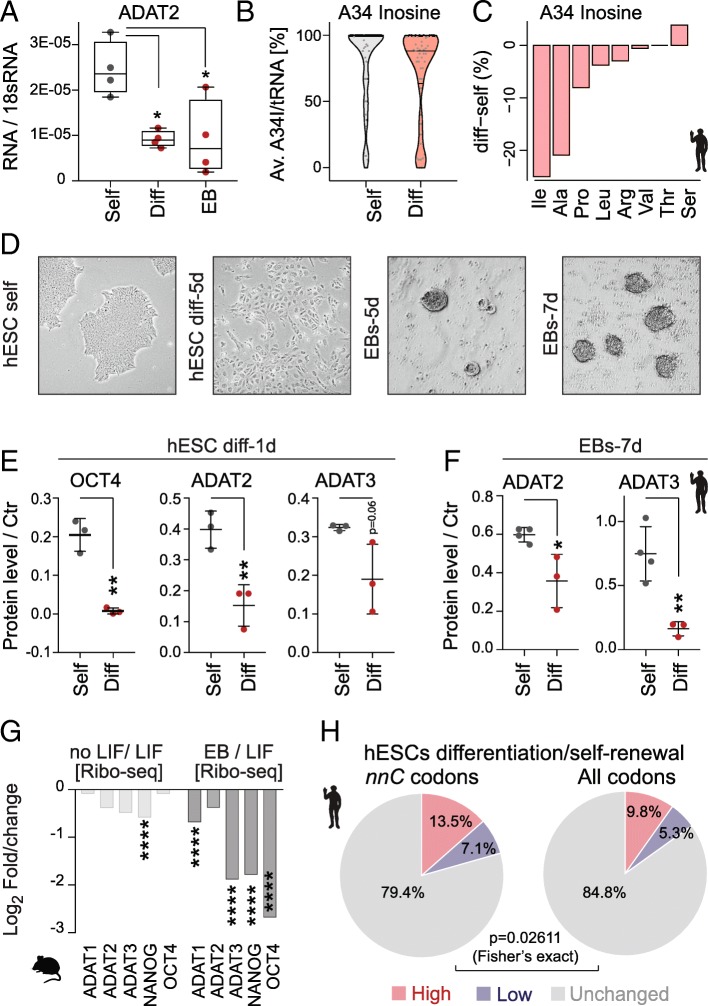
HetADAT-dependent translation in mouse and human ESCs. **a** RT-qPCR confirming downregulation of ADAT2 mRNA levels in differentiated hESCs (Diff) and embryoid bodies (EB) compared to self-renewing hESCs (Self). * *p* < 0.05 one-way ANOVA. **b** Distribution of wobble inosine (A34I) levels per tRNA (> 10 reads) shown as Violin plot with median (black line) and quartiles (dotted line). **c** Differences of inosine levels at A34 in differentiated versus self-renewing hESC per tRNA isotype. **d** Light microscopic image of self-renewing (self) human embryonic stem cells (hESCs), differentiated hESCs for 5 days (5d), and embryoid bodies (EBs) after 5 and 7 days in suspension. **e** Quantification of protein expression using Western blotting of OCT4, ADAT2, and ADAT3 in self-renewing (grey) and differentiating (red) hESCs for 1 day. **p < 0.01 unpaired *t*-test. **f** Quantification of protein levels using Western blotting of ADAT2 and ADAT3 after differentiating hESCs into EBs for 7 days (7d). *p < 0.05; **p < 0.01 unpaired *t*-test. **g** Log_2_ fold change gene translation (Ribo-seq) of indicated proteins in mouse self-renewing ESCs versus ESCs differentiated by removal LIF or grown as embryoid bodies (EB). ****p < 0.0001. **h** Ribo-seq fold changes (diff/self) was compared with RNA-seq fold changes (diff/self). Genes with ribosome occupancy difference two-fold higher (red), two-fold lower (blue) or similar (grey) to the mRNA level difference was identified among genes rich in hetADAT-dependent *nn*C codons (left) and among all genes (right). The hetADAT-dependent group was significantly enriched for genes with altered (higher and lower) ribosome occupancy

The lower frequencies of hetADAT-dependent codons at the A-site of ribosomes in self-renewing ESCs might indicate an enhanced activity of the catalytic complex. Indeed, the mRNA level of ADAT2 was significantly higher in self-renewing hESCs when compared to RA-differentiated cells or EBs (Fig. 6a). To quantify the levels of inosine in hetADAT-dependent tRNAs, we performed tRNA sequencing in self-renewing and differentiated hESCs (Additional file 5: Table S4). We used the fact that inosine will form the strongest bond to cytosine and is therefore reported as a guanine during sequencing. The level of A34I modifications can thus be quantified by measuring the A-to-G substitutions at the anticodon wobble position (Additional file 6: Table S5). The A34I tRNA modification levels were significantly higher in self-renewing (83–86%) than differentiated (60–79%) hESCs (*p* = 0.031; Student’s *t* test) (Fig. 6b). Accordingly, the A34I modification occurred less often in the majority of hetADAT-dependent tRNA isotypes (Fig. 6c). Thus, self-renewing hESCs have higher levels of A34I tRNA modifications than differentiating cells.

Next, we confirmed that also the protein levels of ADAT2 and ADAT3 decreased with differentiation of hESCs (Fig. 6d–f; Additional file 1: Figure S5A-E). ADAT2/3 protein expression was significantly reduced after only 1 day of treatment with retinoic acid (Fig. 6e; Additional file 1: Figure S5D). Downregulation of hetADAT was further confirmed when the hESCs were grown in suspension to induce their differentiation into EBs (Fig. 6f; Additional file 1: Figure S5E). Similarly, translation of mouse ADAT2 and ADAT3 was downregulated upon differentiation, yet the differences were only significant when compared to differentiated EBs (Fig. 6g).

Finally, we asked whether the optimized codon usage in self-renewing ESCs resulted in differential translation of hetADAT-dependent codons. We calculated the total frequency of the eight *nn*C codons that rely on the inosine modification for translation in all genes (Fig. 5b). Then, we selected all coding sequences containing more than 28.4% (top 1%) *nn*C codons and asked whether they showed translational differences in self-renewing versus differentiated hESCs that were independent of mRNA levels. Comparing the two cell states, we found that translation of 13.5% *nn*C-high coding sequences was enhanced and 7.1% repressed (> 2-fold) (Fig. 6h; left panel). This difference in translation of *nn*C-enriched genes was significantly different when compared to all coding sequences (Fig. 6h; right panel). Thus, the differential translation of *nn*C-high coding sequences in self-renewing hESCs cannot be explained by differences in RNA abundance alone.

Together, our data suggest that self-renewing ESCs translate hetADAT-dependent codons with higher efficiency when compared to differentiating ESCs. This difference in hetADAT-dependent codon usage occurred independently of the nature of the differentiation stimulus and was conserved in mice.

## Discussion

Protein synthesis is a fundamental process in all cells, yet recent studies revealed distinct regulatory functions of the mRNA translation machinery in stem cells. Low global protein translation rates are commonly found in adult undifferentiated stem and progenitor cells and are required to maintain a fully functioning stem cell state [61–63]. Mouse embryonic stem cell differentiation also correlates with an overall increase in protein synthesis and enhanced mRNA loading into polyribosomes [64]. One explanation for the differences in protein synthesis might be the expression of distinct tRNA pools that optimize codon usage in different cell states [6]. However, the use of tRNA concentrations to understand codon usage is hampered by the fact that in addition of being crucial adaptors during translation, tRNAs are important regulators of a wide range of biological processes [65]. An alternative method to analyse codon usage is ribosome profiling, which has been applied to various organisms and cell contexts, for example amino acid starvation, oxidative stress, the perturbation of signalling pathways, and embryonic stem cell differentiation [49, 66–69].

Here, we use ribosome profiling to analyse the importance of optimized codon usage in regulating gene expression in different cell states. Our work identifies a codon bias in self-renewing and differentiating stem cells, and we propose that this is driven by both mRNA levels and translational mechanisms. Genes belonging to the same functional category use similar codons characterized by differences in GC content. Since we measured similar GC content enrichment in up- and downregulated genes, our observed differences are unlikely to be directly linked to mRNA degradation. Our data is consistent with those of previous studies in Neurospora, where codon usage strongly correlated with protein and mRNA levels genome-wide but was largely independent of mRNA translation and stability [70].

Thus, GC content is one important factor that influences codon choice during gene expression. The concept that codon optimization is due to the GC content of the underlying genes has been demonstrated before for both specific genes and genome-wide [9, 41, 42]. However, we further propose that the regulation of the protein synthesis machinery through chemically modified tRNAs plays important additional roles in regulating gene expression programmes. We find that codons whose translation depends on hetADAT-modified tRNAs were under-represented at the ribosome A-sites in self-renewing mouse and human ESCs. We provide further evidence that a faster translation of hetADAT codons in self-renewing cells might be facilitated by higher levels of inosines at the tRNA wobble position. While a role for hetADAT in embryonic stem cell fate has not been described before, it is well known that tRNA wobble modifications ensure efficient protein synthesis, maintain protein homeostasis, and promote cell adaptation and survival [71].

The existence of a nuclear (GC content) and a cytoplasmic (translation) mechanism that both determine codon usage during stem cell differentiation raises the question of how they are connected. It is plausible that a cytoplasmic tRNA modification-driven codon bias may help to overcome a GC-content-driven codon usage for cell type-specific mRNAs, thereby enhancing translation of certain groups of mRNAs. However, the functional analyses on how precisely hetADAT regulates codon usage and how this influences gene expression in pluripotent embryonic stem cells is hampered by the fact that ADAT2/3 is essential for cell survival [56]. Knock-down of hetADAT causes cell death in human cells. Over-expression also affects cell survival because ADAT2/3 is a highly mutagenic enzyme [72]. Therefore, ADAT2/3-mediated C to U deamination at position 32 requires the prior formation of 3-methylcytosine in *Trypanosoma brucei* [73]. Thus, increasing the hetADAT levels might not be sufficient to increase inosines specifically at the wobble positions.

Together, we provide evidence for an hetADAT-dependent codon bias in self-renewing embryonic stem cells that might suppress differentiation and lineage commitment.

## Conclusion

In this study, we used RNA-seq and Ribo-seq to decipher transcriptional and translational mechanisms regulating codon bias in self-renewing and differentiating human embryonic stem cells. We revealed that codon usage during stem cell differentiation is regulated at the mRNA levels and during translation. We confirm that codon usage of differentially expressed genes is primarily characterized by genomic GC content. Furthermore, we reveal a novel mechanism based on tRNA modifications that regulate codon usage in pluripotent embryonic stem cells. Translation of codons that depend on the hetADAT-mediated inosine formation in the anticodon loop of tRNAs are under-represented at the ribosome A-site. The reduced stalling of these codons at the A-site implies their enhanced translation in self-renewing embryonic stem cells when compared to differentiated cells. Thus, we reveal that tRNA modifications contribute to optimized codon usage in self-renewing embryonic stem cells.

## Material and methods

### Human embryonic stem cell culture and differentiation

The human embryonic stem cell (hESC) line Hues9 (H9) was obtained from the Wicell Research Institute (Madison, WI). hESCs were maintained in Essential 8 media (Thermo Fisher Scientific) on hESC-qualified Matrigel (Corning)-coated plates at 37 °C with 5% CO_2_. Cultures were dissociated in clumps using 0.5 mM EDTA in PBS every 4 days and media was renewed daily. Differentiating hESCs were cultured in MEF-conditioned KSR media plus 1 μM retinoic acid in DMSO for 5 days, while the control hESC population was maintained either in MEF-conditioned KSR media completed with 4 ng/ml FGF2 or Essential 8 media (Thermo Fisher Scientific). During the course of the experiment, the media were replaced daily. KSR media consists of 85% KO-DMEM (Thermo Fisher Scientific), 15% KO-serum replacement (Life Technologies), 1 mM Glutamax (Thermo Fisher Scientific), 0.1 mM 2-mercaptoethanol (Thermo Fisher Scientific), and 0.1 mM non-essential amino acids (Thermo Fisher Scientific). In the embryoid body experiments, 70% confluent hESCs were dissociated in clumps using 0.5 mM EDTA in PBS and seeded in ultra-low attachment well plates (Corning) maintaining a 1:1 dilution factor. hESCs were cultured in Essential 6 media (Thermo Fisher Scientific) plus 10 μM rock inhibitor (Y-27632) (Stem Cell Technologies Canada) for the first 24 h and for further 4 to 6 days in Essential 6 media (Thermo Fisher Scientific), when samples were collected for qPCR or Western blot experiments.

### RNA, qPCR, and Western blot

Total RNA was extracted using TRIZOL (Thermo Fisher Scientific) according to the manufacturer instructions. Reverse transcription was performed using SuperScript III Reverse Transcriptase (Thermo Fisher Scientific) and random primers (Promega). Quantitative PCR were run using TaqMan probes (Thermo Fisher Scientific) for eukaryotic 18S rRNA (X03205.1), ADAT2 (Hs00699339_m1) CDX2 (Hs01078080_m1), DLX3 (Hs00270938_m1), DLX5 (Hs01573641_mH), DNMT3B (Hs00171876_m1), FOXD3 (Hs00255287_s1), GATA6 (Hs00232018_m1), HOXA1 (Hs00939046_m1), NANOG (Hs02387400_g1), POU5F1 (Hs03005111_g1), PRDM14 (Hs01119056_m1), and TDGF1 (Hs02339497_g1).

Protein extracts used for western blotting were prepared in RIPA buffer (50 mM sodium chloride, 1.0% NP-40, 0.5% sodium deoxycholate, 0.1% SDS, 50 mM Tris, pH 8.0). The antibodies used in the experiments were anti-HSP90 (sc-13119) 1:5000 and anti-OCT3/4 (sc-5279) 1:1000 both from Santa Cruz Biotechnologies and anti-ADAT2 (ab135429) 1:1000 and anti-ADAT3 (ab125514) 1:1000, both from Abcam. The Western blot band intensity was measured using ImageJ.

### Immunofluorescence

For immunofluorescence experiments, hESCs were plated on Matrigel-coated coverslips and cultured as described. At the desired time point, cells were washed in PBS and fixed for 10 min in ice-cold 4% PFA. Cell permeabilization was performed in 0.01% Triton x-100 in PBS for 5 min at room temperature. Coverslips were blocked for 1 h at room temperature using 10% donkey serum in PBS. Anti-ADAT2 (ab135429) 1:100 and anti-ADAT3 (ab125514) 1:100 primary antibodies were incubated overnight in the same blocking solution. Alexa Fluor secondary antibodies (Thermo Fisher Scientific) were used at a dilution of 1:500 in blocking solution. Nuclei were counterstained using DAPI. Images were acquired on a Leica SP8 Confocal microscope and processed.

### Preparation of RNA-seq, Ribo-seq, and tRNA seq libraries

Total RNA from self-renewing and differentiated hESCs (four biological replicates per condition) was extracted using Trizol according to the manufacturer’s instruction. Total RNA was treated with the RiboZero Magnetic Kit (Epicentre, MRZH11124) to remove ribosomal RNA. Libraries for sequencing were prepared using the NEB ultra-directional library prep kit (NEB).

Ribosome-protected RNA was isolated as described before [61, 74]. Briefly, at the indicated time points, H9 cells (4 replicates) were washed with PBS and lysed in 20 mM Tris-Cl (pH 7.4), 150 mM NaCl, 5 mM MgCl2, 1 mM dithiothreitol (DTT) (Sigma), 1% Triton X-100 (Sigma), 25 U ml − 1 of Turbo DNase I (Thermo Fisher Scientific), and 100 μg ml^−1^ of cycloheximide (Sigma). After passing the lysates ten times through a 26-G needle, they were spun down at 13,000 rpm for 10 min. Digestion with RNaseI (100 U μl^−1^, Thermo Fisher Scientific) for 45 min at room temperature was used to produce ribosome mRNA footprints. The RNaseI digestion was inhibited with SuperaseIN (Thermo Fisher Scientific), and lysates were fractionated on a 1 M sucrose cushion by ultracentrifugation at 45,000 rpm in a 70Ti rotor for 9 h at 4 °C. The ribosome mRNA footprints were further purified using Qiazol reagent. Footprints with a length of 26–34 nucleotides were size selected on 15% TBE-urea gel (Thermo Fisher Scientific) and 3′-dephosphorylated with T4 polynucleotide kinase (10 U, NEB). All samples were multiplexed and sequenced on the HiSeq2500 platform (Illumina). The NGS data are up-loaded onto GEO (GSE123611): Bornelöv S, Selmi T, Flad S, Dietmann S, Frye M. Codon usage optimization in pluripotent embryonic stem cells. Datasets. Gene Expression Omnibus.https://www.ncbi.nlm.nih.gov/geo/query/acc.cgi?acc=GSE123611(2019).

For tRNA sequencing, differentiating, and self-renewing, H9 (four replicates per condition) were collected in TRIZOL (Thermo Fisher Scientific) at day 5 of differentiation. Fifty microgrammes of total RNA samples was DNaseI digested (Ambion) at 37 °C for 30 min. The treated samples were then subjected to phenol-chloroform extraction and isopropanol precipitation and resuspended in 1 mM EDTA 0.1 M Tris-HCl (pH 9.0). One microgramme of each replicate was used to generate cDNAs and perform qPCR to confirm differentiation. The remaining sample was heated at 37 °C for 30 min in order to de-aminoacylate tRNAs. Subsequently, samples were heated for 5 min at 80 °C and then loaded on a Novex™ TBE-Urea polyacrylamide gel (Thermo Fisher Scientific). A gel fragment spanning from 50 to 100 nucleotides, according to the Abnova RNA marker low easy, was excised from the gel and resuspended in 400 μl of a solution of 300 mM NaAc pH 5.5, 1.0 mM EDTA, and 0.25% SDS. RNA was extracted by a cycle of 30-min freezing at − 80 °C followed by overnight gentle mixing at room temperature in the same buffer, and subsequently was ethanol precipitated. RNA ends of any tRNA fragments were phosphorylated by treatment with 20 U of T4 Polynucleotide Kinase (NEB) supplemented with ATP (NEB). Following heat inactivation and phenol chloroform extraction, and isopropanol precipitation, samples were subjected to library preparation using the Illumina Truseq small RNA kit (Illumina). Samples were multiplexed and sequenced on the HiSeq4000 platform (Illumina).

### Processing of RNA-seq, Ribo-seq data, and tRNA-seq data

Human RNA-seq and Ribo-seq data were obtained as described above. Mouse RNA-seq and Ribo-seq data with accession numbers SRR315620-SRR315627 and SRR31591-SRR31600 were downloaded from the NCBI short read archive [49]. *Trim_galore!* was used to trim small RNA adapters from the human data (auto-detected under default settings) or polyadenylation sequences from the mouse data (using the parameters “--stringency 1” and “--adapter” with ten “A” followed by multiple “N”). Trimmed reads shorter than 20 nt were discarded. Alignment was done using Tophat2 (v2.1.0) [75]. The RNA-seq reads were aligned directly to the reference genome (hg38 or mm10). Ribo-seq reads were firstly aligned to a set of known rRNA and tRNAs (selected from the UCSC RepeatMasker tracks), followed by alignment of all unmapped reads to the reference genome. An index with known transcripts (Gencode v23 or Gencode vM9) were provided for the genome alignment, and novel splice junctions were permitted [76, 77]. Multi-mapping read alignments were not used.

For tRNA-seq data analyses, *Trim galore!* with “--paired --stringency 6 -a TGGAATTCTCGG -a2 GATCGTCGGACT” was used to trim small RNA adapters and to remove reads shorter than 20 nucleotides. Alignment was done using bowtie [78] with “-n 2 -y -k 1 --nomaqround --allow-contain” to allow for up to two mismatches in the alignment and to report one alignment per read. The reads were aligned to the 430 tRNAs from the high-confidence set in GtRNAdb [79]. Introns were removed, and the “CCA” tail was added before creating the bowtie index.

Modified bases were identified using samtools mpileup with “-BQ 0 -t AD -vuf” to report base coverage per positions. Inosine modifications were identified as positions with an A-to-G substitution at position 33–35. The presence of the expected anticodon in the reference sequence was used to verify that only substitutions at the correct nucleotide were counted. We identified 21 Ala, 7 Arg, 8 Ile, 9 Leu, 9 Pro, 8 Ser, 7 Thr, and 9 Val tRNAs with at least one mapped read supporting the substitution. These are the same eight tRNA isotypes that were expected to be modified. Next, we estimated the overall modification level per sample as the percentage of reads supporting the modification (A-to-G substitution) across all 78 modified tRNAs.

### Meta-gene profile

*DeepTools* were used to quantify the Ribo-seq coverage across the whole reference genome [80]. Each strand was quantified separately, and a blacklist file containing all rRNA, tRNA, snoRNA, snRNA, and miRNA was provided. The bin size was set to 1 and an offset of 12 was used to only consider a single nucleotide corresponding to the “P” site predicted from each read. A meta-gene profile for all protein coding genes was then computed using *computeMatrix* with the parameters “scale-regions -b 500 --unscaled5prime 200 --regionBodyLength 1000 --unscaled3prime 200 -a 500” to define relative coordinates and “--metagene --exonID CDS --transcriptID gene --transcript_id_designator gene_id” to define the coding sequence of each gene. Finally, a trimmed mean was used to exclude the most extreme value at each position.

### Differential mRNA levels and translation

Gene models were downloaded from Gencode (v23 for human and vM9 for mouse). *FeatureCounts* was used to quantify the number of reads per gene, represented by either all annotated exons (RNA-seq) or all annotated coding sequences (Ribo-seq) [81]. Only reads aligning to the sense strand of the gene and with mapping quality of at least 20 was counted. For the paired-end RNA-seq, the additional flags “-B -C” were specified to exclude chimeric reads and/or reads mapping with only one end. Differential mRNA expression and translation analyses were done with *DESeq2* [82]. The heatmaps were created using the R package pheatmap (https://CRAN.R-project.org/package=pheatmap), and the amino acid logo was created using the DiffLogo package [83].

### Determining read periodicity and the position of the ribosome P-site

Ribosome profiling data display 3-nt periodicity, but the reading frame of the 5′ read ends differ between different experiments and even between read lengths within the same experiment. The human data showed the strongest periodicity for reads of length 27–29 (Additional file 1: Figure S1E-F), the 5′ end of those reads were highly enriched for the first codon position, and the most frequent starting position was located 12 positions upstream of the TIS. We therefore decided to base our codon analysis in humans on these three read lengths and to extract read position 12–14 as the P-site codon.

The mouse data showed different dominating reading frames for different read lengths (Additional file 1: Figure S4C). To predict the strongest reading frame for each read length, we again calculated the number of reads starting from each position around the TIS (see Additional file 1: Figure S4D for an example). Next, we counted the most frequent reading frame both per codon (from codon position − 7 to + 32 relative to the TIS) and across the whole region. We excluded read lengths where the reading frame that was most abundant in the highest number of codons was different from the one that was most abundant in total. Furthermore, we required the most abundant reading frame was at least five-fold more abundant than the second most abundant both in terms of the number of codons and in total. This resulted in read lengths 26, 29, 31–36, and 39 being selected for the mouse data. Out of these, read length 39 was later excluded due to having substantially fewer reads and showing poor correlation to the others. Since the highest number of reads started 13 or 14 positions upstream of the TIS (depending if frame 3 or 2 was most abundant), we trimmed one or two nucleotides of the reads in the subsequent analysis so that the P-site would still be located at read position 12–14 similar to the human data.

### Extracting codon counts per position relative to the A-site

The bam file for each sample with uniquely aligned reads was converted to bed format. *Bedtools intersect* was used to select reads with at least 50% overlap to Gencode-annotated coding sequences. Next, the reading frame of the 5′ end of each read was determined using the frame information in the Gencode annotation. If the frame did not agree with the expected reading frame for that read length, the read was discarded. For the mouse data, reads starting with the third or second position of a codon had one or two nucleotides trimmed off the read to put all reads into the same reading frame. Then, nucleotide positions 1–27 were extracted from each read as nine codons, numbered as codon position − 5 to + 3, where 0 corresponded to the A-site. Next, we counted the number of occurrences of each codon per position, and we validated the approach by verifying that codon counts calculated using different read lengths but corresponding to the same codon position (e.g. the P-site) correlated better to each other than to any other codon counts. Furthermore, counts from position − 4 to − 2 and + 1 to + 3 correlated well to the genome-wide distribution of codons in the human and mouse translatomes, whereas counts from the predicted P-site and A-site did not. We therefore concluded that we were counting codons from the correct frame for each included read length for both human and mouse.

### Codon enrichment

The number of codon occurrences were counted separately for each ribosome-protected codon position and converted into a fraction of the total number. Normalized codon counts were obtained by dividing the codon fraction at a specific position by the mean fraction across all nine positions. Significant differences in normalized codon enrichment were calculated using Student’s *t* test using the four biological replicates per cell state. The *p* values were corrected for multiple testing using FDR correction. The figures are showing the signal across pooled replicates.

### GO term analysis

Coding sequences were downloaded from the consensus CDS project. GO relations (go-basic.obo) were downloaded from the Gene Ontology Project (http://www.geneontology.org) and parsed using the Perl module GO::Parser. Relative codon frequencies were calculated per gene and then averaged per GO term. Only GO terms with at least 40 genes were considered. The PCA analysis was performed in *R* using the method *princomp* using the correlation matrix.

## Additional files

Additional file 1: **Figures S1 to S5.** Including figure legends. (PDF 1667 kb)

Additional file 2: **Table S1.** RNA-seq and Ribo-seq data in the four replicates of self-renewing (self) and differentiating (diff) human embryonic stem cells. (XLSX 4135 kb)

Additional file 3: **Table S2.** Gene enrichment analyses (EnrichR) identifies highly expressed genes in tissues derived from all three germ layers using ARCHS4 enrichment tool. (XLSX 11 kb)

Additional file 4: **Table S3.** Distribution of hetADAT targets at ribosome covered codons in (a) differentiated versus self-renewing hESCs. The A-site is highlighted in yellow and (b) differentiated embryoid bodies (EB) versus self-renewing mESCS. The A-site is highlighted in yellow. (XLSX 13 kb)

Additional file 5: **Table S4.** tRNA sequencing in self-renewing and differentiated (5 days) human embryonic stem cells. (XLSX 10 kb)

Additional file 6: **Table S5.** tRNA sequencing to quantify inosine levels in differentiated (diff) and self-renewing (self) human embryonic stem cells. (XLSX 26 kb)

## Data Availability

The sequencing data used in our study have been deposited in NCBI’s Gene Expression Omnibus and are accessible through the GEO accession number GSE123611 [84].
