# Supplementary material for: Codon usage optimization in pluripotent embryonic stem cells
Source: Genome Biol. 2019 Jun 7;20:119. doi: 10.1186/s13059-019-1726-z (PMC6555954; doi:10.1186/s13059-019-1726-z)
Supplement: Supplementary file 1 — Figures S1 to S5. Including figure legends. (PDF 1667 kb) [file 13059_2019_1726_MOESM1_ESM.pdf]

Fig S1

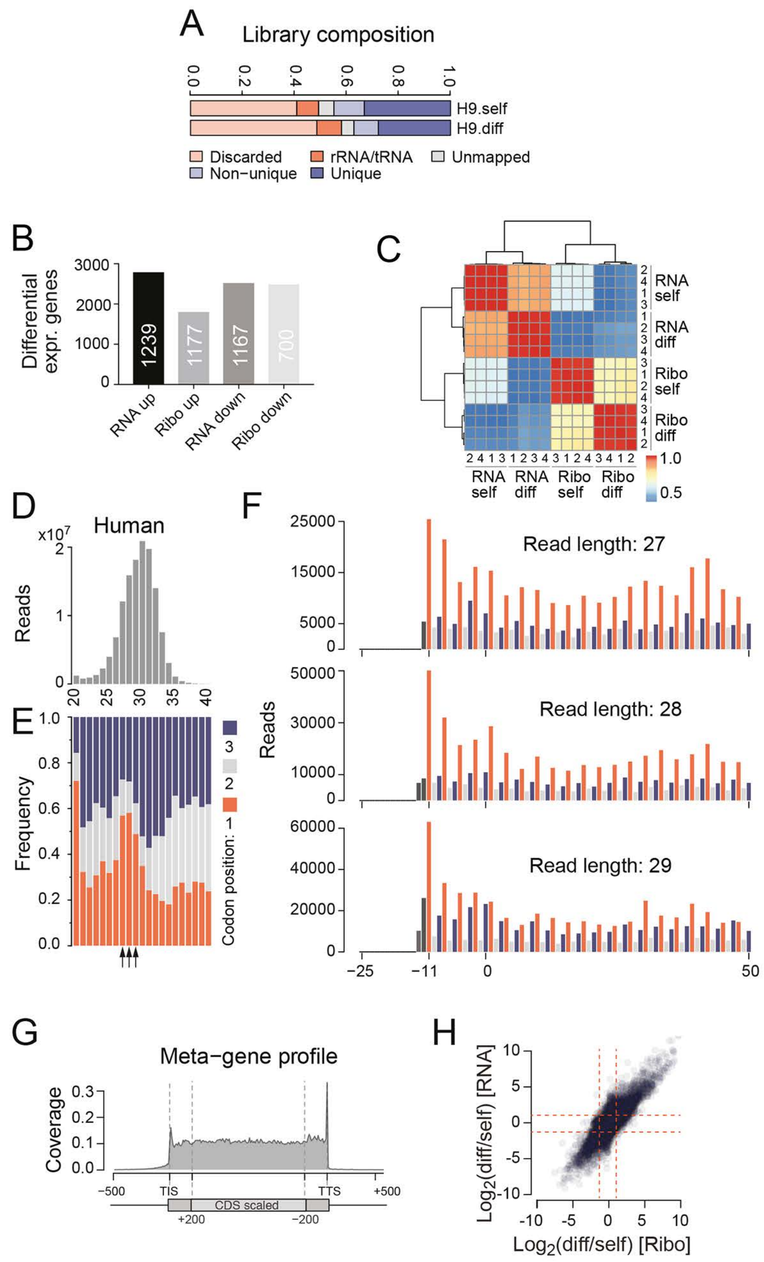

Figure S1. Quality check of ribosome profiling data. (A) Library composition of Ribo-seq dataset. (**B**) Number of differentially expressed genes (padj<0.05; $\log_2$ FC>2 or FC<-2) in RNA- and Ribo-seq datasets. (**C**) Correlation plot between replicates and datasets (RNA-seq and Ribo-seq) in self-renewing (self) and differentiating (diff) hESCs. (**D, E**) Read length distribution (**D**) and reading frame (**E**) of the 5' end of the reads. (**F**) High triplet-periodicity near the start codon (position 1-3) of aligned reads with lengths of 27 to 29 nucleotides. (**G**) Metagene profile of aligned reads over the coding sequence (CDS) and the adjacent 5’ and 3’ untranslated regions. The first and last 200 nucleotides of the CDS are shown unscaled and the remaining CDS was scaled to equal length for all genes. TIS: translation initiation site. TTS: translation termination site. (**H**) Correlation between differentially expressed (RNA-seq) and translated (Ribo-seq) genes.

Fig S2

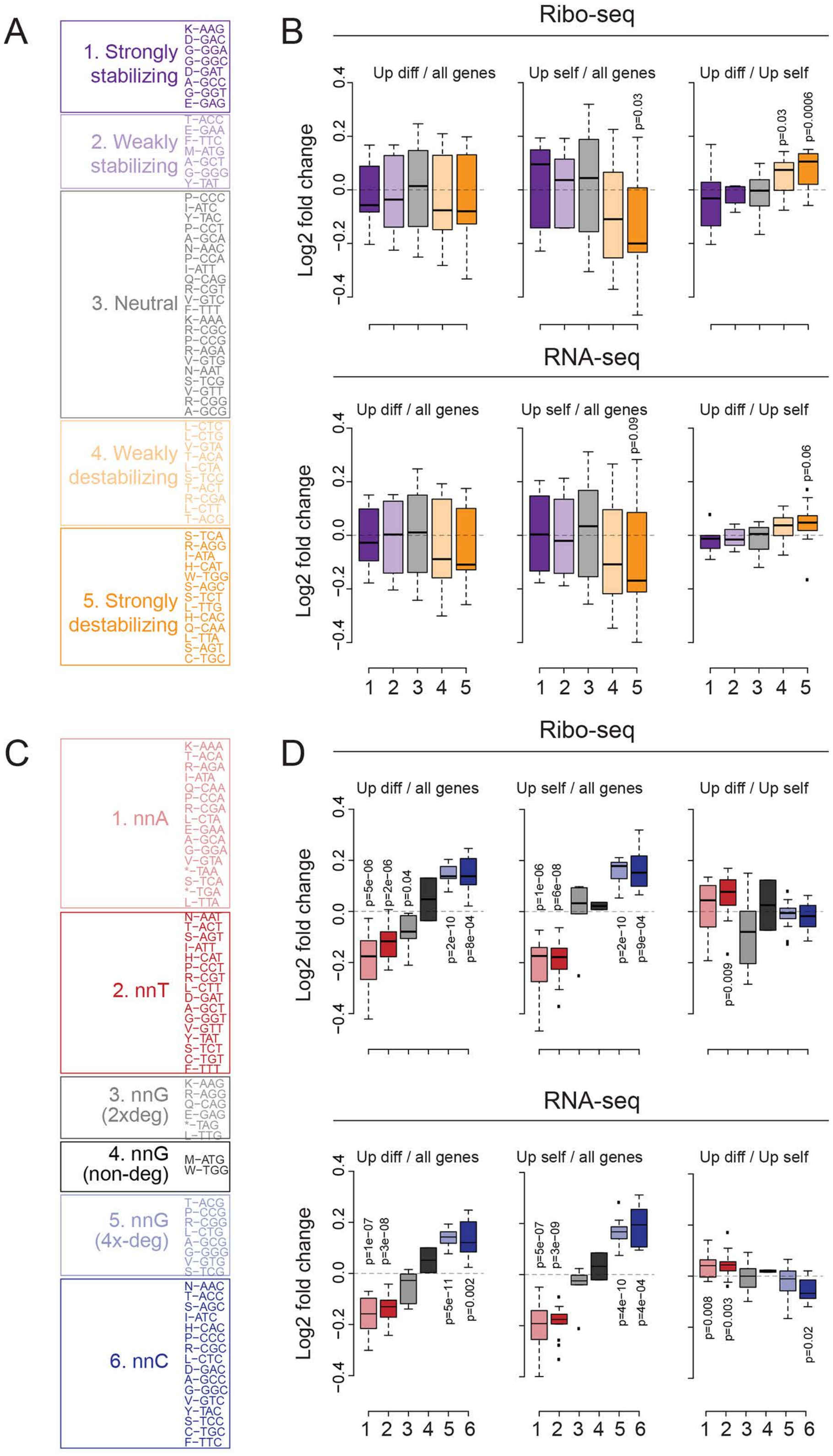

Figure S2. Optimal codons and GC-content contribute to mRNA levels. (A, B) Codons were classified according to their stabilizing or de-stabilizing effect on mRNAs (**A**) and their enrichment measured in up-regulated genes in self-renewing (self) and differentiating (diff) human embryonic stem cells (hESCs) using Ribo-seq or RNA-seq datasets (**B**). (**C**, **D**) Codon enrichment by the third nucleotide and degeneracy (**C**) in up-regulated genes in self and diff hESCs using Ribo-seq or RNA-seq datasets. The righthand columns (**B**, **D**) compare the difference of stabilizing or de-stabilizing codons (**B**) or codon composition (**D**) between up-regulated RNAs in diff and self hESCs.

Fig S3

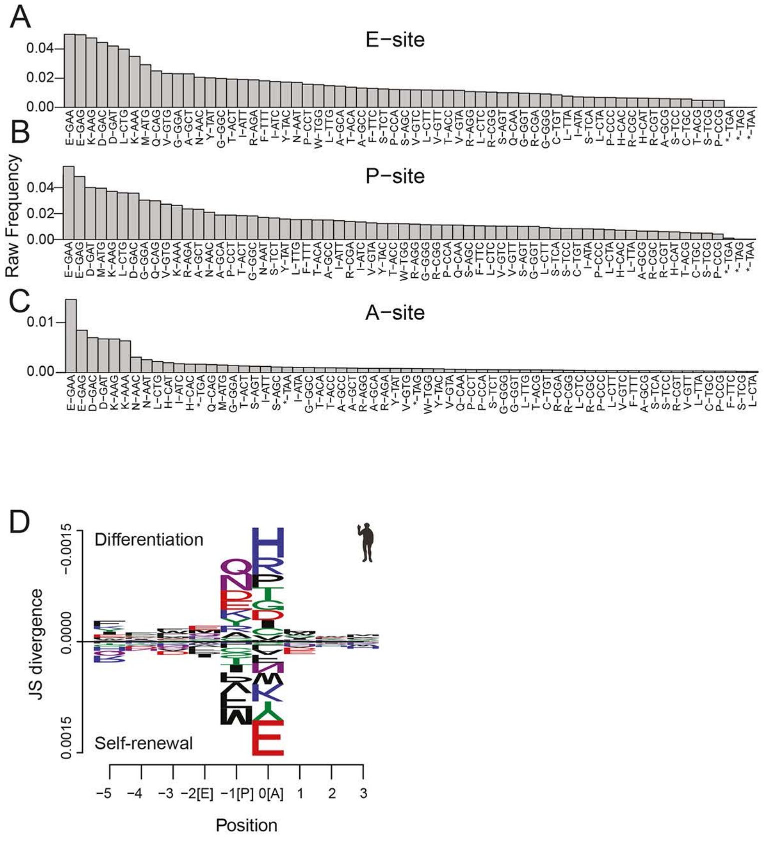

Figure S3. Codon and amino acid frequencies. (A-C) Ratio of raw codon frequency in differentiating versus self-renewing human embryonic stem cells (four pooled replicates) at the ribosome E- (**A**), P- (**B**), and A-(**C**) sites. (**D**) DiffLogo analyses showing the difference in normalized amino acid occupancy between differentiating and self-renewing hESCs at the E, P, and A positions and the flanking positions (-5..-3, +1..+3) covered by the ribosome.

Fig S4

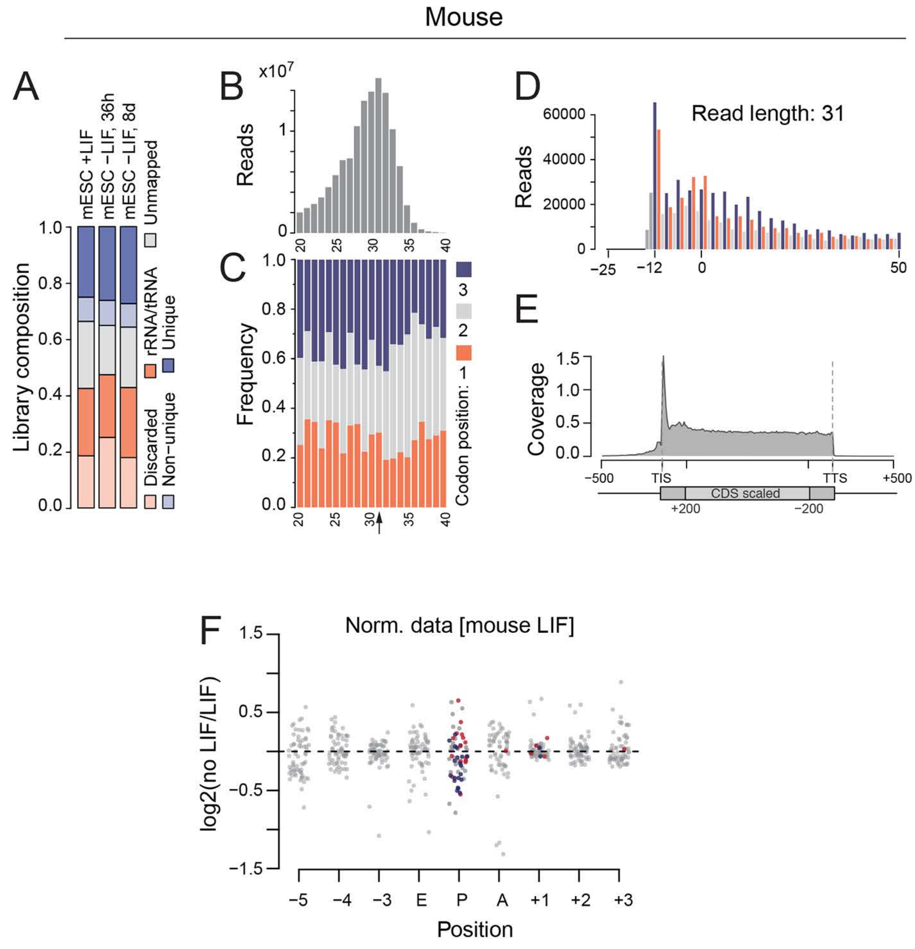

Figure S4. Quality check of mouse ribosome profiling data. (A) Library composition of Ribo-seq dataset. (**B, C**) Read length distribution (**B**) and reading frame (**C**) of the 5' end of the reads. (**D**) Example of triplet-periodicity near the start codon (position 1-3) of aligned reads with lengths of 31 nucleotides. Read lengths 26, 29 and 31 to 36 were used for the analyses. (**E**) Metagene profile of aligned reads over the coding sequence (CDS) and the adjacent 5' and 3' untranslated regionsThe first and last 200 nucleotides of the CDS are shown unscaled and the remaining CDS was scaled to equal length for all genes. TIS: translation initiation site. TTS: translation termination site. (**F**) Log_2 fold-change of normalized codon usage in mouse differentiated (removal of LIF) versus self-renewing embryonic stem cells (mESCs). Blue and red dots represent the significantly changed codon shown in figure 3H.

Fig S5

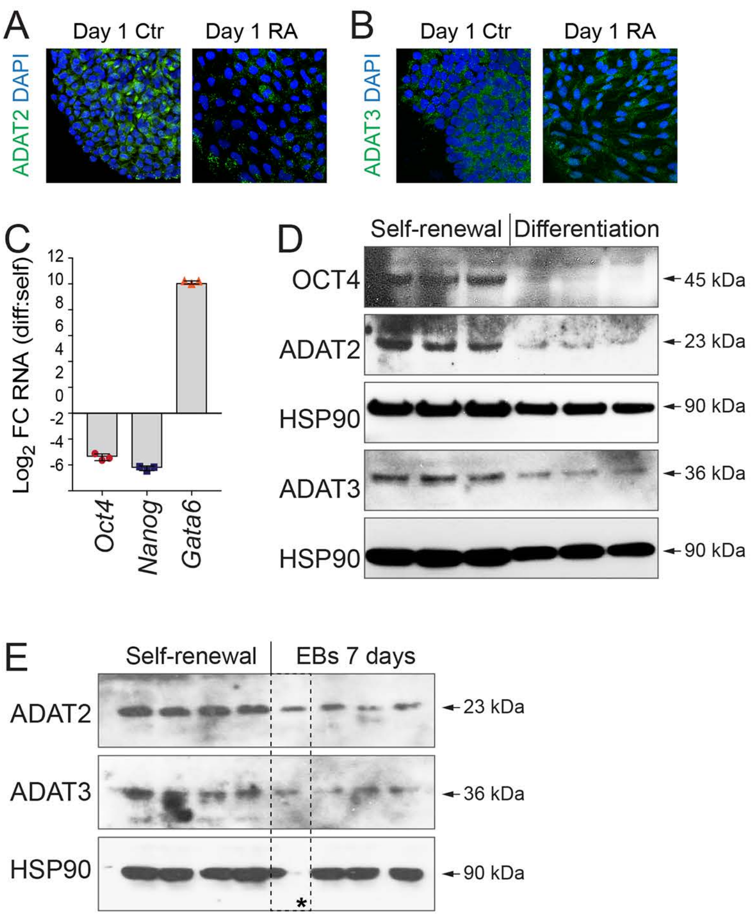

Figure S5. ADAT2 expression in hESCs. (A, B) Immunofluorescence staining for ADAT2 (**A**) and ADAT3 (green) (**B**) in control (ctr) (left hand panels) and retinoic acid (RA)-induced (right hand panels) hESCs for one day. DAPI (blue) counterstains nuclei. (**C**) Log_2 fold change in RNA levels of the indicated genes measured by qPCR. (**D**) Western Blot to quantify expression of the indicated proteins (shown in Figure 6E) in self-renewing and differentiated hESCs. (**E**) Western Blot to quantify expression of the indicated proteins (shown in Figure 6F) in self-renewing hESCs and hESCs differentiated into embryoid bodies (EBs). Asterisk: Excluded from the quantification.
